# Phytochemical Profiling of *Allium subhirsutum* L. Aqueous Extract with Antioxidant, Antimicrobial, Antibiofilm, and Anti-Quorum Sensing Properties: In Vitro and In Silico Studies

**DOI:** 10.3390/plants11040495

**Published:** 2022-02-11

**Authors:** Mejdi Snoussi, Emira Noumi, Hafed Hajlaoui, Lamjed Bouslama, Assia Hamdi, Mohd Saeed, Mousa Alreshidi, Mohd Adnan, Ayshah Al-Rashidi, Kaïss Aouadi, Siwar Ghannay, Ozgur Ceylan, Vincenzo De Feo, Adel Kadri

**Affiliations:** 1 Department of Biology, College of Science, University of Hail, P.O. Box 2440, Ha’il 2440, Saudi Arabia; emira_noumi@yahoo.fr (E.N.); saeedmicrobiology@gmail.com (M.S.); mousa.algladi@gmail.com (M.A.); drmohdadnan@gmail.com (M.A.); ayshah.a@hotmail.com (A.A.-R.); 2 Laboratory of Genetics, Biodiversity and Valorisation of Bioressources, High Institute of Biotechnology, University of Monastir, Monastir 5000, Tunisia; 3 Laboratory of Bioressources: Integrative Biology and Recovery, High Institute of Biotechnology, University of Monastir, Monastir 5000, Tunisia; 4 Research Unit Valorization and Optimization of Resource Exploitation (UR16ES04), Faculty of Science and Technology of Sidi Bouzid, Campus University Agricultural City, University of Kairouan, Sidi Bouzid 9100, Tunisia; bio.hafedh@gmail.com; 5 Laboratory of Bioactive Substances, Center of Biotechnology of Borj Cedria (CBBC), BP 901, Hammam Lif 2050, Tunisia; lamjed.bouslama@gmail.com; 6 Laboratoire de Développement Chimique Galénique et Pharmacologique des Médicaments, Faculté’ de Pharmacie, Université de Monastir, Monastir 5000, Tunisia; hamdiessia@gmail.com; 7 Department of Chemistry, College of Science, Qassim University, Buraidah 51452, Saudi Arabia; k.aouadi@qu.edu.sa (K.A.); s.ghannay@qu.edu.sa (S.G.); 8 Department of Chemistry, Faculty of Science of Monastir, University of Monastir, Monastir 5019, Tunisia; 9 Ula Ali Kocman Vocational School, Mugla SitkiKocman University, Mugla 48147, Turkey; ozgceylan@hotmail.com; 10 Department of Pharmacy, University of Salerno, Via Giovanni Paolo II, 132, Fisciano, 84084 Salerno, Italy; 11 Department of Chemistry, College of Science and Arts in Baljurashi, Albaha University, Albaha 65731, Saudi Arabia; lukadel@yahoo.fr; 12 Department of Chemistry, Faculty of Science of Sfax, University of Sfax, B.P. 1171, Sfax 3000, Tunisia

**Keywords:** *Allium subhirsutum* L., phytochemistry, antioxidant, antimicrobial, antibiofilm, anti-quorum sensing, pharmacokinetics, toxicological prediction

## Abstract

The present study was the first to evaluate the phytochemical composition, antioxidant, antimicrobial, antibiofilm, and anti-quorum sensing potential of *Allium subhirsutum* L. (hairy garlic) aqueous extract through in vitro and in silico studies. The phytochemical profile revealed the presence of saponins, terpenes, flavonols/flavonones, flavonoids, and fatty acids, particularly with flavonoids (231 ± 0.022 mg QE/g extract), tannins (159 ± 0.006 mg TAE/g extract), and phenols (4 ± 0.004 mg GAE/g extract). Gas chromatography–mass spectrometry (GC–MS) analysis identified 15 bioactive compounds, such as 5-hydroxymethylfurfural (37.04%), methyl methanethiolsulfonate (21.33%), furfural (7.64%), beta-D-glucopyranose, 1,6-anhydro- (6.17%), 1,6-anhydro-beta-D-glucofuranose (3.6%), trisulfide, di-2-propenyl (2.70%), and diallyl disulfide (1.93%). The extract was found to be non-toxic with 50% cytotoxic concentration higher than 30,000 µg/mL. The investigation of the antioxidant activity via DPPH (2, 2-diphenyl-1-picrylhydrazyl) and FRAP (IC_50_ = 1 μg/mL), ABTS (2,2′-azino-bis(3-ethylbenzothiazoline-6-sulfonic acid); IC_50_ = 0.698 ± 0.107 μg/mL), and β-carotene (IC_50_ = 0.811 ± 0.036 mg/mL) was assessed. Nevertheless, good antimicrobial potential against a diverse panel of microorganisms with bacteriostatic and fungistatic effect was observed. Quorum sensing inhibition effects were also assessed, and the data showed the ability of the extract to inhibit the production of violacein by the mutant *C. violaceum* strain in concentration-dependent manner. Similarly, the biofilm formation by all tested strains was inhibited at low concentrations. In silico pharmacokinetic and toxicological prediction indicated that, out of the sixteen identified compounds, fourteen showed promising drug ability and could be used as lead compounds for further development and drug design. Hence, these findings support the popular use of hairy garlic as a source of bioactive compounds with potential application for human health.

## 1. Introduction

In modern medicine, many bacteria and their associated infections are considered the major challenge in public health worldwide, causing annually 35,000 deaths in the United States and about 33,000 deaths in European Union [1,2]. Furthermore, the treatments with synthetic drugs are often associated with higher side effects and cannot be tolerated by some people at high doses. Contrary, the exploration of herbs and plant-based products remains important and one of the most applied pharmacological alternatives for the prevention and the treatment of several pathologies as well as a large number of illnesses due to their safety, affordability, and availability [3,4]. Their richness in bioactive molecules such as polyphenols and biochemical components of phytomedicines (alkaloids, flavonoids, phenolics, carotenoids, polysaccharides, lactones, and tannins, …) gives them potent therapeutic benefits, allowing them to be a potential escort to the development of new drug candidates [5,6,7,8,9,10,11,12]. They have a powerful action in reducing the threat of numerous chronic diseases caused by free radicals, destroying the immune system, and translating into serious oxidative stress and oxygen (O_2_) detoxification [12,13].

Besides that, the spread of infection disease, as well as the emergence of multidrug-resistant bacteria and fungi, is increased recently due to the failure of chemotherapy and the indiscriminate or frequently uses of antibiotics, and their inhibition by alternative agents become an urgent need. Therefore, attention is now being shifted towards natural active components isolated from herbs along with phytomedicine which shares high antioxidant and antimicrobial effects with low cost and high efficiency. As a result, they have been used to avoid various curable infection diseases as the WHO has pointed out that ‘‘no action today means no cure tomorrow”. 

In this respect, *Allium subhirsutum* L. is a perennial plant that belongs to the garlic family. Recent literature conducted by our team reported on the ethno-pharmacological use of bulbs for therapeutic claims, including antioxidant, anti-inflammatory, and anticancer claims, as well as those relating to the inhibition of tumor angiogenesis in a murine model of skeletal metastases [14,15]. Based on an evaluation of our previous study, the phytochemical analysis of this plant has only been assessed by HR-LCM for methanolic extract; the results from this indicate that it contains various phytoconstituents, particularly polyphenols and flavonoids, along with several other bioactive compounds [15,16,17,18,19,20,21]. Whole plant is used in the popular medicine in Sardinia (Italy) due to its anti-hemorrhoidal, blood pressure regulation, and purifying action [22]. In addition, hairy garlic buds and leaves are cut into small pieces and eaten raw or used as flavoring agent in cooked dishes and salads [23]. It is also a good ingredient in the traditional Turkish flat bread called yufka [24]. Few studies have described the phytochemical composition of the essential oil and organic extracts from *A. subhirsitum* plant organs (bulbs, flowers, and leaves). Our team has demonstrated that methanolic extract from hairy garlic (bulbs) helps with antioxidant, anti-inflammatory, and anticancer claims, and can inhibit tumor angiogenesis in a murine model of skeletal metastases [16,20]. Nencini and colleagues reported the antioxidant activities of aged 15% aqueous ethanol extract from Italian *A. subhirsutum* leaves, flowers, and bulbs [21]. Emir and colleagues reported the cholinesterases and tyrosinase inhibition activities of Turkish *A. subhirsutum* (methanolic extract from bulbs and aerial parts) [18].

Hence, the present work was the first to quantitatively analyze the phytochemical constituents of the aqueous extract obtained from of *A. subhirsutum* bulbs. Additionally, the determination of its antioxidant, antimicrobial, anti-quorum sensing, antibiofilm, cytotoxicity, and antiviral properties with the aqueous extract was also carried out. Moreover, to screen potential drugs/molecules from the above extract, pharmacokinetics and toxicological prediction were also performed.

## 2. Results

### 2.1. Phytochemical Composition of A. subhirsutum L. Aqueous Extract

The aqueous extract from the hairy garlic bulbs was screened for the presence of different classes of biomolecules. Results showed that hairy garlic aqueous extract is a rich source of saponins, terpens, flavonols/flavonones, flavonoids, and fatty acids. 

Table 1 summarizes the tentative identification of phytoconstituents in the tested extract by using the GC–MS technique. Fifteen compounds, belonging to different chemical classes, were identified. 5-hydroxymethylfurfural (37.04%), methyl methanethiolsulfonate (21.33%), furfural (7.64%), beta-D-glucopyranose, 1,6-anhydro- (6.17%), and 1,6-anhydro-beta-D-glucofuranose (3.6%) were the dominant compounds, followed by trisulfide, di-2-propenyl (2.70%), and diallyl disulfide (1.93%). Their chemical formula and molecular weight are listed in Table 1.

The chromatogram obtained showed, respectively, the peaks of the identified molecules with their chemical structure (Supplementary Material S1).

### 2.2. Biological Activities of A. subhirsutum L. Aqueous Extract

#### 2.2.1. Cytotoxicity Evaluation

Results obtained using the MTT [3-(4,5-dimethylthiazol-2-yl)-2,5-diphenyltetrazolium bromide] coloremetric method against the African green monkey kidney cell lines (VERO cell lines) showed that hairy garlic aqueous extract was not toxic as the 50% cytotoxic concentration (CC_50%_) was higher than 30,000 µg/mL. 

#### 2.2.2. Antimicrobial Activities

The hairy garlic aqueous extract was tested against a large collection of Gram-positive and Gram-negative bacteria, yeasts, molds, and coxsakievirus B3 (CVB3), and herpes simplex virus type 2 (HSV-2). Results of the antiviral activity showed that *A. subhirsutum* aqueous extract was not active against the two tested virus (CVB3) and (HSV-2). 

Results of the antibacterial and antifungal activities of the hairy garlic aqueous extract using both disc diffusion and microdilution assays are summarized in Table 2.

The mean diameter of the growth inhibition zone (mm ± SD) varied from 6.00 ± 0.00 to 15.66 ± 0.57. Using the scheme proposed by Parveen et al. (2010), our extract had low (1–6 mm) to high (11–15 mm) activity against all tested Gram-positive and Gram-negative bacteria. Most of tested microorganisms were more sensitive to the tested extract as compared to the standard antibiotic used (ampicillin). Using the microdilution assay, MIC values ranged from 6.25 to 12.5 mg/mL and concentrations around 50–100 mg/mL could kill the tested microorganisms (MBCs values). The MBC/MIC ratio ranged from 4 to 8, highlighting the bacteriostatic activity of the tested extract on all tested microorganisms. This extract was also active against yeast and mold strains at varying degrees. The Candida vaginalis strain was the most sensitive to hairy garlic extract with a mean diameter of about (15.66 ± 0.57 mm) in the growth inhibition zone. MIC values ranged from 1.62 to 6.25 mg/mL and MFCs values ranged from 25 to 50 mg/mL. The tested aqueous extract exhibited fungistatic activity against all *Candida* spp. strains tested (MFC/MIC ratio > 4). 

#### 2.2.3. Antioxidant Activities

The quantitative analysis of tannins, phenols, and flavonoids demonstrated that hairy garlic contains large quantities of flavonoids (231 ± 0.022 mg QE/g extract), followed by tannins (159 ± 0.006 mg TAE/g extract) and phenols (4 ± 0.004 mg GAE/g extract). The results of the free radical scavenging activities of *A. subhirsutum* L. aqueous extract, as compared to ascorbic acid and butylated hydroxyl-toluene (BHT), are reported in Table 3. The concentrations needed for scavenging 50% of radicals (IC_50%_) using hairy garlic aqueous extract were as follows: 1 mg/mL for DPPH and FRAP assays (respectively), (0.698 ± 0.107) mg/mL for the ABTS test, and (0.811 ± 0.036) mg/mL for the β-carotene experiment. 

#### 2.2.4. Anti-Quorum Sensing and Antibiofilm Activities

**Figure 1:**
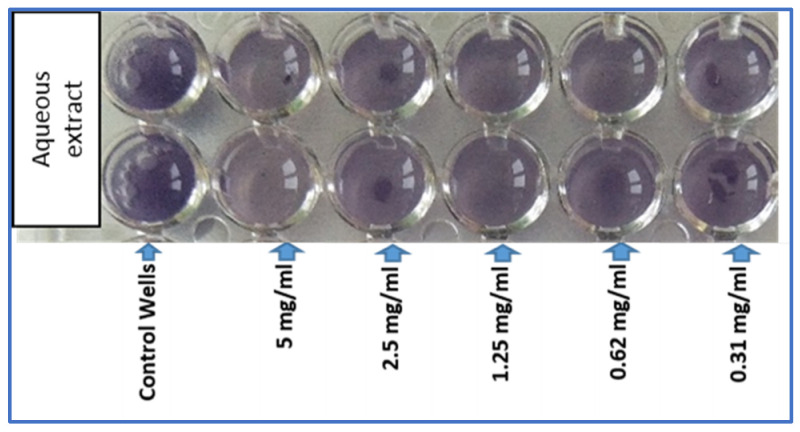
Qualitative method for the determination of the effect of different concentrations of *A. subhirsutum* aqueous extract on violacein production by *C. violaceum* CV026.

The ability of *A. subhirsutum* aqueous extract to inhibit the production of violacein, a pigmented molecule secreted by *C. violaceum* when bacterial concentration is reached (quorum sensing), was tested using the microtiter plate assay (mutant-*C*. *violaceum* CV026) and the Petri dishes test using Luria–Bertani agar medium (wild type *C. violaceum* ATCC 12472). The results showed that the tested extract was able to inhibit the production of violacein by the mutant *C. violaceum* strain in a concentration-dependent manner (Figure 1). In fact, the percentage of violacein production was inhibited by (24.05 ± 0.68)% at 2.5 mg/mL and about (37.43 ± 0.85)% at 5 mg/mL. No inhibition of violacein production was recorded at low concentrations of hairy garlic extract (1.25 and 0.625 mg/mL). 

Using LB Petri dish agar plates, the tested extract was able to inhibit the production of violacein by the wild-type starter strain (*C. violaceum* ATCC 12472). Furthermore, the diameter of growth inhibition zone ranged from (8 ± 1) mm at 1.25 mg/mL to (13 ± 0.5) mm at 5 mg/mL. All these data are summarized in Table 4.

In addition, two virulence properties controlled by the quorum-sensing system in the *P. aeruginosa* PAO1 strain (swarming and swimming) were inhibited at varying degrees when different concentrations of hairy garlic aqueous extract were used. In fact, the percentage of the inhibition of swarming activity ranged from (8.93 ± 0)% at 50 µg/mL to (23.66 ± 0.5)% at 100 µg/mL. Furthermore, the swimming activity was reduced by (13.67 ± 1)% at 100 µg/mL of the hairy garlic aqueous extract (Table 5). 

The antibiofilm activity of the tested extract was experimented on four bacteria and two *Candida* spp. strains at different concentrations, ranging from MIC/16 to MIC (from 0.312 to 10 mg/mL). Table 6 summarizes the percentage of biofilm inhibition when different concentrations of extract were used. 

Interestingly, at an MIC value of (10 mg/mL), the percentage of biofilm inhibition depended on the tested strain and increased from (12.18 ± 1.24)% for *L. monocytogenes* ATCC 7644, (18.50 ± 1.35)% for *E. coli* ATCC 25922, (32.97 ± 2.56)% for *S. typhi* ATCC 14028, and (56.21 ± 2.55)% for *S. aureus* ATCC 25923. The fungal biofilm was also inhibited at low concentrations. In fact, 1.25 mg/mL of hairy garlic extract inhibited the ability of *C. albicans* ATCC 10239 and *C. tropicalis* ATCC 13803 by (5.20 ± 0.62)% and (15.23 ± 2.52)%, respectively. The highest inhibition was recorded at an MIC value of (10 mg/mL of aqueous extract) with a percentage of inhibition at about (62.48 ± 5.50)% for *C. albicans* ATCC 10239 and (54.81 ± 4.08)% for *C. tropicalis* ATCC 13803. Based on Duncan’s multiple-range test, there was no significant difference between the percentage of biofilm inhibition of the two *Candida* strains tested at 10 mg/mL.

#### 2.2.5. Pharmacokinetic Properties and Toxicity Profile Prediction

During the time of the preclinical analysis trial in drug discovery and development, the assessment of absorption, distribution, metabolism excretion, and toxicity (ADMET) are very crucial for attractive molecules to possess the best chance to become an effective drug. Hence, based on ADME analysis, the identified phytocompounds from *A. subhirsutum* water extract were predicted for their pharmacokinetics, drug-likeness, and medicinal chemistry friendliness using the SwissADME web tool. As shown in (Table 7), all selected phytoconstituents did not violate the Lipinski’s rule of five; therefore, they seem to be passed orally with suitable bioavailability scored at 0.55. They exhibited high gastrointestinal (GI) absorption with eight compounds being able to cross the blood-brain–barrier (BBB) permeant; this revealed that they have low to no central nervous system (CNS) side effects. Only ten compounds were found in the substrate for permeability glycoprotein (P-gp), meaning that they possess very little chance to efflux out of the cell. Moreover, compounds **1**–**7** did not inhibit all the tested cytochrome P450 isoenzymes which played a fundamental role in the biotransformation of drugs through O-type oxidation reactions. 

The AMES toxicity and hepatotoxicity of the parameters of hERG I/II inhibitors were evaluated using the pkCSM online server which predicted whether the designed new molecules were toxic. Based on the predictive results (Table 7), except the compound 1, none of the others were expected to present any toxicity problems. 

**Figure 2:**
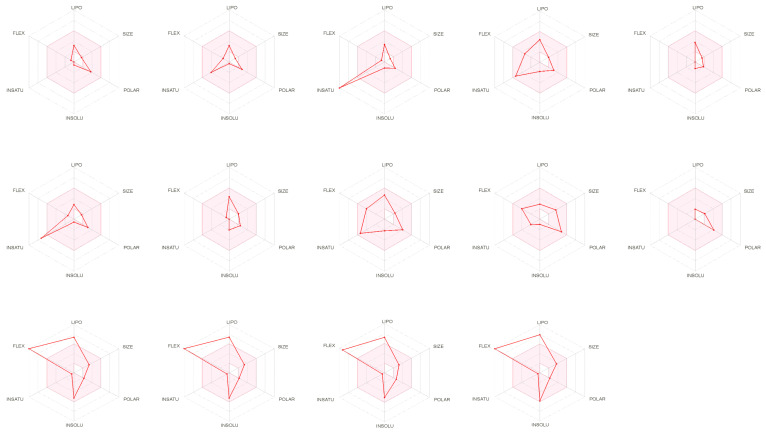
Bioavailability radar of identified compounds based on the physicochemical indices ideal for oral bioavailability. LIPO, lipophilicity: −0.7 < XLOGP3 < þ 5; SIZE, molecular size: 150 g/mol < molecular weight < 500 g/mol; POLAR, polarity: 20 Å2 < TPSA (Topological Polar Surface Area) < 130 Å2; INSOLU, insolubility: 0 < Log S (Insolubility in water: ESOL) < 6; INSATU, Insaturation: 0.25 < Fraction of carbons in the sp3 hybridization < 1; FLEX, flexibility: 0 < the number of rotatable bonds < 9. The colored zone is the suitable physicochemical space for oral bioavailability.

An estimation of drug-likeness properties based on bioavailability radar (Figure 2) remains a powerful tool to understand identified compounds, as well as their lipophilicity, size, polarity, solubility, saturation, and flexibility behaviors. In addition, as shown in Figure 2, most of the identified compounds fit totally in the pink area, signifying their good predicted oral bioavailability.

**Figure 3:**
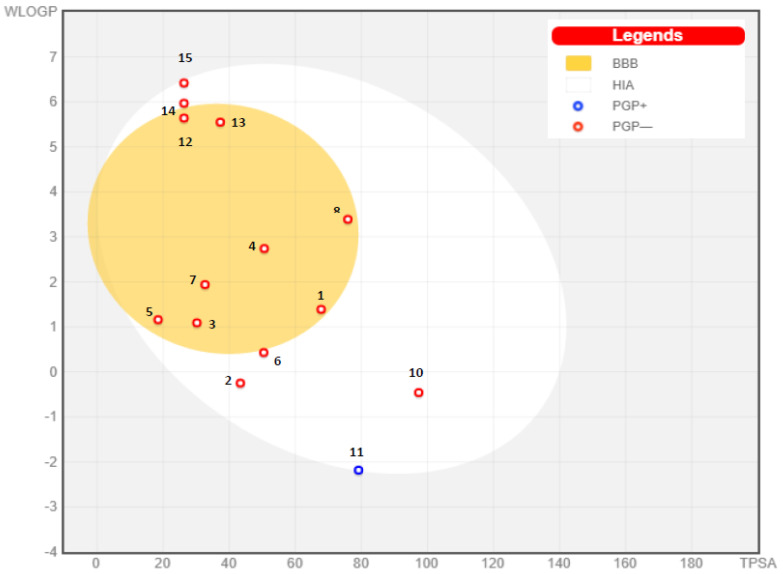
(BOILED–Egg) model of the selected compound. The names of the compounds are listed in Table 1.

To know more about both passive gastrointestinal absorption (HIA) and the BBB penetrating effect as a function of the position of the molecules in the WLOGP-versus-TPSA referential, the boiled egg model (Figure 3) has been established for the top ADME compounds. The results clearly indicate that compounds 2, 6, 10, 11, 14, and 15 were in the white zone, thus indicating the high probability of being passively absorbed by the gastrointestinal tract with only 11 appearing as a blue point, which was predicted as a substrate of the PGP+. On the other hand, compounds 1, 3, 4, 5, 7, 8, 12, and 13 were in the in the yolk region, which reflects their high probability to permeate through BBB to access CNS, and also appeared in the red point, suggesting that the substrate of the p-glycoprotein is actively effluxed by PGP+. 

## 3. Discussion

In the present study, we were the first to investigate the chemical composition of *A. subhirsutum* (bulbs) aqueous extract using the GC–MS technique. We reported the identification of 15 bioactive compounds belonging to different known classes of bioactive molecules. In fact, 5-hydroxymethylfurfural (37.04%), methyl methanethiolsulfonate (21.33%), furfural (7.64%), trisulfide, di-2-propenyl (2.70%), and diallyl disulfide (1.93%) were identified and compared to published data, as reviewed in Table 8.

By using the LC-MS technique, we reported the identification of 16 small peptides (5 dipeptides and 11 tripeptides), and 25 phytoconstituents dominated by sebacic acid, 11alpha-acetoxykhivorin, cepharanthine, methyl gamboginate, hexadecasphinganine, 4-Oxomytiloxanthin, and linolenoyl lysolecithin from hairy garlic methanolic extract [16]. In 2020, Sut and colleagues [17] reported the identification of several bioactive compounds from the aerial parts and bulbs of *A. subhirsutum* L. (ethanolic extract) collected from Padova (Italy). The ethanolic extract from the dried pulverized bulbs was dominated by alliin (29.51 ± 0.04 mg/g), allicin (26.40 ± 0.02 mg/g), gamma-glutamyl (S)-allylcysteine (10.97 ± 0.02 mg/g), luteolin (20.19 ± 0.03 mg/g), methoxy quercetin isomer (22.19 ± 0.02 mg/g), glucosyl gallate (21.61 ± 0.0 mg/g), and *N*-trans-feruloyl-tyramine (41.88 ± 0.10 mg/g). In addition, Emir and colleagues [18] reported the identification of 30 phenolic compounds from the aerial parts of *A. subhirsutum* L. methanolic extract by using LC-ESI-MS/MS technique. The major phenolic acids identified were: benzoic acid (39.2 ± 2.83; 146.7 ± 0.95) µg/mL; 3-hydroxybenzoic acid (518.6 ± 1.98; 430.1 ± 2.63) µg/mL; 4-hydroxybenzoic acid (976.7 ± 3.44; 314.2 ± 1.78) µg/mL; *p*-coumaric acid (1700.8 ± 1.52; 1042.4 ± 2.97) µg/mL; vanillic acid (903.8 ± 2.31; 621.6 ± 1.87) µg/mL; gallic acid (44.5 ± 0.93; 52.3 ± 2.21) µg/mL; ferulic acid (787.2 ± 1.18; 1352.0 ± 3.16) µg/mL; and genistein (130.6 ± 1.42; 159.3 ± 2.76) µg/mL, respectively, for the methanolic extract from air-dried and powdered bulbs and aerial parts [18]. In 2018, Küçük and colleagues [19] were the first to study the headspace volatiles from *A. subhirsutum* L. crashed bulbs from Turkey using the gas chromatography–mass spectrometry technique. They reported the identification of six compounds namely allyl methyl disulfide (41.0%), diallyl disulfide (20.7%), dimethyl sulfide (15.3%), methyl (methylthiol) methyl disulfide (2.0%), methyl trans-propenyl disulfide (1.9%), and allyl methyl trisulfide (1.4%). 

Most of the identified compounds in hairy garlic aqueous extract were previously described in the composition of different *Allium* species, as summarized in Table 9. 

In terms of the phytochemical composition, our results were similar to those obtained by Saoudi and colleagues in 2021 [20]. This team quantified the total phenols and flavonoids in *A. subhirsutum* cloves (methanolic extract and oil) collected from Sfax (Tunisia). They founded that the methanolic extract was a rich source of tannins, phenols, and flavonoids (41.7 ± 3.4), as compared to the corresponding oil. The difference in the phytochemical composition can be attributed to the solvent used. In fact, using the same plant species from the same origin (Hail, Saudi Arabia) and methanol as eluent, our team reported different amounts of these classes of compounds. The origin of plant samples also affects the quantity of phenolic and flavonoid contents. In fact, Emir and colleagues [18] reported that the total phenolic content of *A. subhirsutum* methanolic extract from the aerial parts was about (13.3 ± 1.7 mg of quercetin equivalent/g of dry plant extract) as compared to bulbs (15.8 ± 0.9 mg of quercetin equivalent/g of dry plant extract). On the other hand, the quantity of flavonoids was higher in bulbs (5.7 ± 0.8 mg of quercetin equivalent/g of dry plant extract) as compared to the aerial parts (3.6 ± 0.3 mg of quercetin equivalent/g of dry plant extract). Interestingly, Nencini and colleagues [21] confirmed that phenolics and flavonoids decreased in the aged hydroethanolic extract (up to 20 months) of different organs from *A. subhirsutum*. In fact, the polyphenol levels in aged hairy garlic extract were about (0.39 ± 0.01 mg GAE/g) in bulbs, (1.22 ± 0.01 mg GAE/g) in leaves, and (1.09 ± 0.02 mg GAE/g) in flowers.

No scientific report discussed the anti-quorum sensing and the antibiofilm activities of *A. subhirsutum* plant extracts. Interestingly, our extract was able to modulate the secretion of violacein by both *C. violaceum* mutant and wild type. The same extract inhibited the swarming and swimming motility mode of *P. aeruginosa* PAO1 in a concentration-dependent manner. Previous reports have focused on extracts from *A. cepa* and *A. sativum* plant species. Results showed that sulfur compounds (Ajoene, Iberin) from garlic and flavonoids (Quercetin) from red onion modulated the production of bioluminescence in *Vibrio harveyi*, violacein in *C. violaceum*, and pyocyanin/proteases/elastases and swarming motility in *P. aeruginosa* [38]. *Allium cepa* (95% methanolic) extract was also able to inhibit the production of the green pigment (Pyocyanin) by *P. aeruginosa* PAO1 and to reduce its motility by reducing swimming, twitching, and swarming abilities [39]. The same extract was able to reduce the production of violacein on agar medium with a mean diameter of about 10 mm in the growth inhibition zone and to prevent the formation of biofilm formed by *P. aeruginosa* (Isolate PA14) using the tube assay method [40]. 

*A. sativum* (methanolic and ethanolic) extracts were able to inhibit the biofilm formed by six pathogenic bacteria including *S. aureus*, *Bacillus cereus*, *Streptococcus pneumoniae*, *P. aeruginosa*, *E. coli*, and *K. pneumoniae* in a concentration-dependent manner [41]. Bhatwalkar and colleagues in 2019 [42] reported that the fresh garlic extract at 4% significantly inhibited the biofilm formed by Shiga-toxin-producing *E. coli* (STEC) strains. More recently, Caputo et al., [43] reported the antibiofilm activities of two landraces (Irsina and Contursi) of *A. ampeloprasum* var. *holmense* from south Italy (methanolic extract from bulbs and aerial parts) which were tested against *Listeria monocytogenes* ATCC 7644, entero-hemorrhagic *E. coli* DSM 8579, *P. aeruginosa* DSM 50071, *Pectobacterium carovotorum* DSM 102074, and *S. aureus* ATCC 25923.

The reported biological properties of the tested extract can be attributed to the presence of many phytocompounds with promising activities. In fact, it is well documented that the main identified compound in hairy garlic extract, 5-hydroxymethylfurfural, possessed antioxidant and antiproliferative activities [44], as well as anti-ischemic and anti-tyrosine enzyme effects, improving blood rheology and affecting the role of glycyrrhizin metabolism [45]. It has been demonstrated that this compound (5-hydroxymethylfurfural) can protect human vein epidermal cell against H_2_O_2_ and glucose and improve acute liver injury in mice [46].

Additionally, methyl methanethiolsulfonate which is produced in different amounts by *Alliaceae* members is an anti-oomycete agent with antimicrobial and antimutagenic characters [47]. In addition, this sulfur compound is known to inhibit colon tumor [48]. More recently, Vijayakumar and Ramanathan [49] demonstrated that 5-hydroxymethylfurfural showed interesting anti-quorum sensing and antibiofilm activities against *C. violaceum*, *Streptococcus pyogenes*, *S. mutans*, *S. aureus*, and *S. epidermidis*. In fact, at 100 μg/mL, this compound inhibited the production of violacein by 87% and reduced the biofilm formation by *S. mutans* and *S. epidermidis* strains by 86% and 79%, respectively. The streptococcal ad staphylococcal biofilm formation was also inhibited with high percentage at 125 μg/mL (up to 83% for *S. pyogens*, and 82% for *S. aureus*). 

Furfural, a natural furan occurring compound, is known to possess antityrosinase and antimicrobial activities against *Bacillus subtilis* with an antimicrobial zone between 16 and 20 mm at 1.4 µM, MIC/MBC values about 0.027 µM [50]. This compound (furfural) was also active against *Salmonella* bacteria with an antimicrobial zone less than 15 mm at concentrations ranging from 0.35 to 1.4 µM, and MIC/MBC values of about 0.029 µM and 0.121 µM, respectively [50]. In addition, the identified sulfur compounds diallyl disulfide and diallyl trisulfide are known to be potent phytoconstituents for the prevention and treatment of several human diseases, such as endocrine system diseases, cardiovascular diseases, neurological diseases, infectious diseases, and cancerous diseases [51,52,53,54].

Similarly, hexadecenoic acid identified in hairy garlic aqueous extract is known to exhibit anti-tumoral, antimicrobial, antioxidant, anti-cholesterol, and anti-inflammatory properties [36,55,56]. Moreover, the identified fatty acid methyl ester (hexadecenoic acid, methyl ester; 9,12-octadecadienoic acid, methyl ester; octadecanoic acid, methyl ester) are known to possess antibacterial, antifungal, and antioxidant activities [57,58].

## 4. Material and Methods

### 4.1. Plant Material Sampling and Extract Preparation

**Figure 4:**
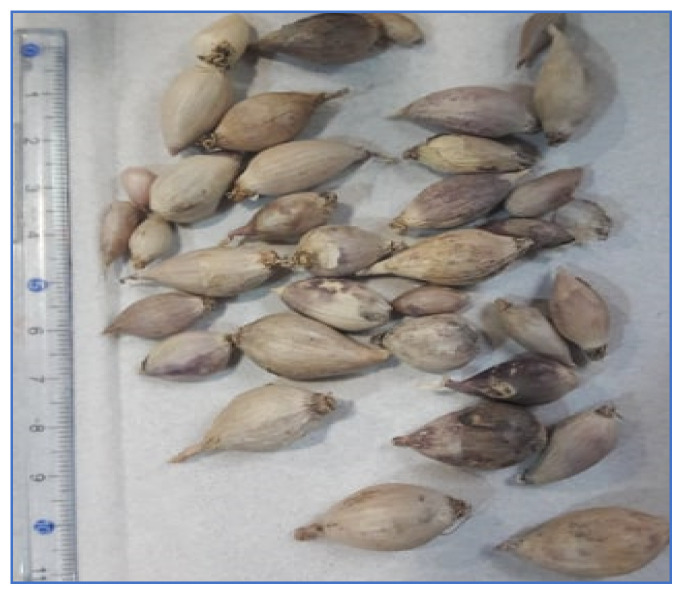
*Allium subhirsutum* L. (bulbs) purchased from a local market in Hail region.

Hairy garlic bulbs (Figure 4) were purchased from a local market in 2020 from Hail region (Saudi Arabia). A voucher specimen (AN03) was deposited at the herbarium in the Department of Biology (College of Science, University of Hail, Hail, Kingdom of Saudi Arabia). Briefly, 40 g of bulbs were macerated in 400 mL of distillated pure water at room temperature for 48 h and re-extracted three times using the same procedure. The yield expressed in percentage was calculated using the following equation (Equation (1)):

Yield (%) = (W1 × 100)/W2(1)

where W1 is the weight of extract after the evaporation of solvent and W2 is the dry weight of the sample.

The obtained extract was filtered, and water was lyophilized by using Millirock Technology apparatus (Kingston, NY, USA) to yield amorphous powder. The yield of extraction was about 27.366 ± 0.152%.

### 4.2. Phytochemical Profiling of Hairy Garlic Aqueous Extract

A Shimadzu Nexis GC-2030 Gas Chromatograph system equipped with a QP2020 NX Mass Spectrometer was used to identify the bioactive compounds in *A. subhirsutum* L. aqueous extract. Helium was used as a carrier gas in the constant flow mode at 1 mL/min. The initial temperature of the column was 70 °C. It was maintained at this temperature for 2 min and was then gradually increased by 10 °C up to 280 °C. The oven temperature was raised up to 280 °C at the increased rate of 5 °C/min and maintained for 9 min. The injection port temperature was 250 °C and the helium flow rate was 1 mL/min. The ionization voltage was 70 eV. Separation was achieved by a RTSvolatile column about 30 m long. A Quadrupole Mass Detector was employed to detect compounds when they were vented from the column. The temperature of the detector was 300 °C. Using MS data libraries, such as WILEY8.LIB and NIST08, the spectrum was analyzed, and compounds were identified.

The presence of alkaloids, flavonoids, terpenoids, tannins, saponins, steroids, proteins, aminoacids, and cardiac glucosides in hairy garlic aqueous extract was quantified using protocols previously described by Sofowora [59], Trease and Evans [60], and Adetuyi and Popoola [61]. 

The total phenols content was estimated using the Folin–Ciocalteu method [62]. The total contents of tannins and flavonoids were estimated using the techniques described by Broadhurst and Jones [63] and Benariba et al. [64], respectively.

### 4.3. Biological Activities

#### 4.3.1. Cytotoxicity Evaluation

The cytotoxicity of the obtained extract was evaluated based on the same protocol carried out by Snoussi et al. [65].

#### 4.3.2. Antimicrobial Activities

The obtained aqueous extract was tested for its ability to act as an antibacterial agent, as previously described by Haddaji et al. [66], against twelve bacteria including four type strains (*Escherichia coli* ATCC 35218, *Pseudomonas aeruginosa* ATCC 27853, *Proteus mirabilis* ATCC 29245, and *Klebsiella pneumoniae* ATCC 27736) and eight clinical strains (*Proteus mirabilis*, *Staphylococcus sciuri*, *Streptococcus pyogens*, *Pseudomonas aeruginosa*, multi-drug resistant *Staphylococcus aureus*, *Enterobacter cloacae*, *Stenotrophomonas paucimobilis*, and *Acinetobacter baumannii*). Three *Candida* strains (*C. albicans* ATCC 10231, *C. vaginalis*, and *C. albicans*), one *C. neoformans* ATCC 14116 strain, and two *Aspergillus* species (*A. fumigatus* ATCC 204305, *A. niger*) were also used. 

The disk diffusion assay was used for the determination of the diameter of the growth inhibition zone estimated on agar medium. The microdilution assay was used for the determination of the minimal inhibitory concentration (MICs) and the minimal bactericidal/fungicidal concentrations (MBC/MFC). To interpret the mean diameter of the growth inhibition zone obtained on agar media, the scheme proposed by Parveen et al. [67] was used. The results of the (MBC/MIC) and (MFC/MIC) ratios were interpreted using the scheme proposed by Gatsing et al. [68]. 

Coxsakievirus B-3 (CVB3) and herpes simplex virus type 2 (HSV-2) were used to test the antiviral potential of the aqueous extract using the procedure described by Alreshidi et al. [69]. 

#### 4.3.3. Antioxidant Activities

The ability of hairy garlic (aqueous extract) to scavenge the DPPH (free-radical 2, 2-diphenyl-1-picrylhydrazyl) and ABTS (2,2′-azino-bis(3-ethylbenzothiazoline-6-sulfonic acid) radicals were calculated using the protocol described by Chakraborty and Paulraj [70]. The β-carotene method described by Ikram et al. [71] was used. The ferric-reducing power was estimated following the method described by Bi et al. [72].

### 4.4. Evaluation of Anti-Quorum Sensing Activity

The inhibition of violacein production was assayed using two starter strains: *Chromobacterium violaceum* ATCC 14272 and CV026, as previously described by Noumi et al. [73]. On the Lauria–Bertani agar plate, activity was interpreted as moderate when the inhibition zone < 10 mm and potent when zone > 10 mm) [74].

The effect of the *A. subhirsutum* aqueous extract to inhibit the motility of the *Pseudomonas aeruginosa* PAO1 strain was tested on 0.3% agar medium (swimming motility) and 0.5% agar medium (swarming motility), as described by Alreshidi et al. [69]. 

#### Anti-Biofilm Activity

The antibiofilm activity of hairy garlic aqueous extract was tested against six reference strains obtained from American Type Culture Collection (ATCC, Manassas, Virginia) strains: *Staphylococcus aureus* ATCC 25923, *Enterococcus faecalis* ATCC 19433, *Escherichia coli* ATCC 25922, *Pseudomonas aeruginosa* ATCC 27853, *Salmonella typhimurium* ATCC 14028, and *Candida albicans* ATCC 10239. The effect 1, 1/2, 1/4, 1/8, and 1/16 MIC concentration value on the biofilm-forming ability of the tested microorganisms was tested using a microplate biofilm assay [75]. 

The percentage of biofilm inhibition (expressed in percentage) was calculated using the following equation (Equation (2)):

Inhibition of biofilm formation (%) = [(OD_Control_ − OD_Sample)_/ODControl] × 100 (2)

where OD_Control_ is the optical density of the control and OD_Sample_ is the optical density of the sample.

### 4.5. ADMET Profile

The pharmacokinetics and the toxicity profiles of the identified molecules were predicted using a SwissADME online server (http://www.swissadme.ch/, accessed on 19 January 2022) and ProTox-II webserver (http://tox.charite.de/tox/, accessed on 19 January 2022) [76,77,78,79]. 

### 4.6. Statistical Analysis

The average values of three replicates were calculated using the SPSS 25.0 statistical package for Windows. Differences in the means were calculated using Duncan’s multiple-range tests for means with a 95% confidence interval (*p* ≤ 0.05).

## 5. Conclusions

Overall, we are the first to report the identification of several small peptides and bioactive molecules in the aqueous extract from *A. subhirsutum* L. using the GC–MS technique. The results obtained allowed the presence of flavonoids (231 ± 0.022 mg QE/g extract), followed by tannins (159 ± 0.006 mg TAE/g extract) and phenols (4 ± 0.004 mg GAE/g extract). Interestingly, several phytocompounds with high biological activities were identified, mainly including 5-hydroxymethylfurfural (37.04%), methyl methanethiolsulfonate (21.33%), furfural (7.64%), beta-D-glucopyranose, 1,6-anhydro- (6.17%), 1,6-anhydro-beta-D-glucofuranose (3.6%), trisulfide, di-2-propenyl (2.70%), and diallyl disulfide (1.93%). These compounds can act independently or synergistically, suggesting their potential application for the treatment of several chronic diseases. Moreover, our results showed significant antioxidant (IC_50_ values; DPPH 1 mg/mL; ABTS 0.698 ± 0.107 mg/mL; β-carotene 0.811 ± 0.036 mg/mL; and FRAP 1 mg/mL) and antimicrobial properties, as well as antibiofilm properties (up to 62.48 ± 5.50 % against *C. albicans* ATCC 10239, and 56.21 ± 2.55% against *S. aureus* ATCC 25923). The tested extract was also able to inhibit the production of violacein by 37.43 ± 0.85% at 5 mg/mL. Swarming and swimming motility in *P. aeruginosa* PAO1 was inhibited by 23.66 ± 0.5% and 13.67 ± 1%, respectively. Our computational study on the major identified compounds revealed acceptable oral bioavailability of the extract, which can be useful in future bioassay studies. Finally, further study on the isolation of significant molecules in vivo studies is strongly recommended in order to evaluate the effectiveness of the isolates for the desired activity.

## Figures and Tables

**Table 1:** Phytochemical composition of aqueous extract of *A. subhirsutum* L. (bulbs) using the GC–MS technique.

N°	Identified Compound Name	RT [min]	Area (%)	Molecular Weight (g/mol)	Formula
1	Methyl methanethiolsulfonate	1.462	21.33	126.20	C_2_H_6_O_2_S_2_
2	Propanoic acid, 2-oxo-, methyl ester	2.201	1.29	102.09	C_4_H_6_O_3_
3	Furfural	3.131	7.64	96.08	C_5_H_4_O_2_
4	Diallyl disulfide	6.725	1.93	146.27	C_6_H_10_S_2_
5	2,4,5-trimethyl-1,3-dioxolane	7.681	2.48	116.16	C_6_H_12_O_2_
6	5-hydroxymethylfurfural	8.857	37.04	126.11	C_6_H_6_O_3_
7	1H-azonine, octahydro-1-nitroso-	9.206	4.09	156.23	C_8_H_16_N_2_O
8	Trisulfide, di-2-propenyl	10.093	2.70	178.34	C_6_H_10_S_3_
9	Beta-D-fructofuranosyl alpha-D-glucopyranoside	11.731	4.40	342.30	C_12_H_22_O_11_
10	Beta-D-glucopyranose, 1,6-anhydro-	12.463	6.17	288.25	C_12_H_16_O_8_
11	Beta-D-glucofuranose, 1,6-anhydro-	13.638	3.60	162.14	C_6_H_10_O_5_
12	Palmitic acid, methyl ester	17.342	3.32	270.45	C_17_H_34_O_2_
13	n-hexadecanoic acid	17.664	1.46	256.42	C_16_H_32_O_2_
14	9,12-octadecadienoic acid, methyl ester	18.974	1.32	294.47	C_19_H_34_O_2_
15	Octadecanoic acid, methyl ester	19.270	1.24	298.5	C_19_H_38_O_2_

**Table 2:** Growth inhibition zone, as well as MIC, MBC, and MFC/MIC values obtained for all microorganisms tested using disc diffusion and microdilution assays tested using hairy garlic aqueous extract.

Code	Strains	*A. subhirsutum* L. Aqueous Extract (10 µL/disc; 100 mg/mL)				Ampicillin (10 µL/disc; 50 mg/mL)
		Mean ± SD * (mm)	MIC	MBC	MBC/MIC	Mean ± SD (mm)
B_1_	*E. coli* ATCC 35218	6.00 ± 0.00 ^fB^	12.5	50	4	7.00 ± 0.00 ^d^^A^
B_2_	*P. aeruginosa* ATCC 27853	11.67 ± 0.57 ^dA^	6.25	50	8	7.33 ± 0.57 ^dB^
B_3_	*P. mirabilis* ATCC 29245	7.00 ± 0.00 ^eA^	12.5	100	8	6.33 ± 0.57 ^dA^
B_4_	*K. pneumoniae* ATCC 27736	6.00 ± 0.00 ^fA^	12.5	100	8	6.66 ± 0.57 ^dA^
B_5_	*P. mirabilis*	6.00 ± 0.00 ^fB^	12.5	100	8	21.00 ± 1.00 ^aA^
B_6_	*S. sciuri*	6.00 ± 0.00 ^fB^	12.5	100	8	7.00 ± 0.00 ^dA^
B_7_	*S. pyogens*	15.66 ± 0.57 ^aA^	6.25	50	8	16.00 ± 1.73 ^bA^
B_8_	*P. aeruginosa*	6.00 ± 0.00 ^fA^	12.5	100	8	6.66 ± 0.57 ^dA^
B_9_	*S. aureus* MDR	15.66 ± 0.57 ^aA^	6.25	50	8	7.33 ± 0.57 ^dB^
B_10_	*E. cloacae*	13.33 ± 0.57 ^cA^	6.25	50	8	6.66 ± 0.57 ^dB^
B_11_	*S. paucimobilis*	6.00 ± 0.00 ^fB^	12.5	100	8	7.66 ± 0.57 ^dA^
B_12_	*A. baumannii*	14.33 ± 0.57 ^bA^	6.25	50	8	13.33 ± 0.57 ^cB^
**Code**	**Strains**	***A. subhirsutum* L. Aqueous Extract** **(10 µL/disc; 100 mg/mL)**				**Amphotericin B** **(10 µL/disc; 10 mg/mL)**
		Mean ± SD * (mm)	**MIC**	**MFC**	**MFC/MIC**	**Mean ± SD (mm)**
Y_1_	*C. albicans* ATCC 10231	6.66 ± 0.57 ^cB^	3.12	50	16	22.66 ± 1.15 ^aA^
Y_2_	*C. neoformans* ATCC 14116	6.33 ± 0.57 ^cB^	3.12	50	16	15.33 ± 0.57 ^bA^
Y_3_	*C. vaginalis*	15.66 ± 0.57 ^aA^	1.56	25	16	6.66 ± 0.57 ^dB^
Y_4_	*C. albicans*	6.00 ± 0.00 ^cB^	6.25	50	8	12.33 ± 0.57 ^cA^
M_1_	*A. fumigatus* ATCC 204305	10.33 ± 0.57 ^bB^	(−)	(−)	(−)	15.00 ± 1.00 ^bA^
M_2_	*A. niger*	6.00 ± 0.00 ^cA^	(−)	(−)	(−)	6.00 ± 0.00 ^dA^

* Inhibition zone around the discs impregnated with *A. subhirsutum* L. aqueous extract expressed as the mean of three replicates (mm ± SD). SD: standard deviation. MIC: minimal inhibitory concentration (mg/mL). MBC: minimal bactericidal concentration (mg/mL). MFC: minimal fungicidal concentration (mg/mL). ^a–f,A,B^: Each value represents the average of 3 repetitions. The means followed by the same letters are not significantly different at *p* = 0.05 based on Duncan’s multiple-range test. Small letters are used to compare aqueous extract and antibiotic means between different strains, while capital letters are used to compare means between aqueous extract and antibiotics for the same strain. (−): Not tested.

**Table 3:** Antioxidant activities of aqueous extract of *A. subhirsutum* L. (bulbs), as compared to ascorbic acid and BHT.

Test Systems	Hairy Garlic Extract	(BHT)	(AA)
Total flavonoids content (mg QE/g extract)	231 ± 0.022	−	−
Total tannins content (mg TAE/g extract)	159 ± 0.006	−	−
Total phenols content (mg GAE/g extract)	4 ± 0.004	−	−
DPPH IC_50_ (mg/mL)	1 ^a^	0.023 ± 3 × 10^−4 b^	0.022 ± 5 × 10^−4 b^
ABTS IC_50_ (mg/mL)	0.698 ± 0.107 ^a^	0.018 ± 4 × 10^−4 b^	0.021 ± 0.001 ^b^
β-carotene IC_50_ (mg/mL)	0.811 ± 0.036 ^a^	0.042 ± 3.5 × 10^−3 b^	0.017 ± 0.001 ^c^
FRAP IC_50_ (mg/mL)	1 ^a^	0,05 ± 0.003 ^c^	0.09 ± 0.007 ^b^

BHT: butylated hydroxytoluene, AA: ascorbic acid. The letters (^a–c^) indicate a significant difference between the different antioxidant methods according to Duncan’s test (*p* < 0.05). (−): Not tested.

**Table 4:** Violacein inhibition anti-quorum sensing activities of *A. subhirsutum* aqueous extract. (−): No activity.

Test	*A. subhirsutum* L. Aqueous Extract (mg/mL)			
	5	2.5	1.25	0.625
Violacein inhibition (%)	37.43 ± 0.85	24.05 ± 0.68	(−)	(−)
Anti-quorum sensing activity (mm)	13 ± 0.5	10 ± 1	8 ± 1	(−)

**Table 5:** Anti-swarming and anti-swimming activities of *A. subhirsutum* extracts.

Tests	100	75	50
Swarming inhibition (%)	23.66 ± 0.5	16.96 ± 1	8.93 ± 0
Swimming inhibition (%)	13.67 ± 1	(−)	(−)

Concentration is expressed as µg/mL; (−): no activity; %: percentage.

**Table 6:** Anti-biofilm results (inhibition %) of *A. subhirsutum* L. aqueous extract tested against Gram-positive and Gram-negative bacteria and yeast strains.

Microorganisms Tested	Concentration Used	*A. subhirsutum* L. Aqueous Extract
*S. aureus* ATCC 25923	MIC = 10 mg/mL	56.21 ± 2.55 ^b^
	MIC/2 = 5 mg/mL	20.50 ± 1.78 ^b^
	MIC/4 = 2.5 mg/mL	2.82 ± 0.13 ^c^
*L. monocytogenes* ATCC 7644	MIC = 10 mg/mL	12.18 ± 1.24 ^e^
*E. coli* ATCC 25922	MIC = 10 mg/mL	18.50 ± 1.35 ^d^
*S. typhi* ATCC 14028	MIC = 10 mg/mL	32.97 ± 2.56 ^c^
	MIC/2 = 5 mg/mL	18.49 ± 1.84 ^c^
	MIC/4 = 2.5 mg/mL	5.24 ± 0.32 ^b^
*C. albicans* ATCC 10239	MIC = 10 mg/mL	62.48 ± 5.50 ^a^
	MIC/2 = 5 mg/mL	35.40 ± 4.25 ^a^
	MIC/4 = 2.5 mg/mL	15.23 ± 2.52 ^a^
*C. tropicalis* ATCC 13803	MIC = 5 mg/mL	54.81 ± 4.08 ^b^
	MIC/2 = 2.5 mg/mL	17.95 ± 2.20 ^c^
	MIC/4 = 1.25 mg/mL	5.20 ± 0.62 ^b^

Each value represents the average of 3 repetitions. The means followed by the same letters are not significantly different at *p* = 0.05 based on Duncan’s multiple-range test. Small letters are used to compare the means between strains for each concentration of aqueous extract.

**Table 7:** ADMET properties of the identified compounds.

Entry	Compounds *													
	1	2	3	4	5	6	7	8	10	11	12	13	14	15
Physicochemical Properties/Lipophilicity/Drug-likeness														
Molecular Weight	126.20	102.09	96.08	146.27	116.16	126.11	156.23	178.34	288.25	162.14	270.45	256.42	294.47	298.50
Num. heavy atoms	6	7	7	8	8	9	11	9	11	19	18	21	21	21
Num. arom. heavy atoms	0	0	5	0	0	5	0	0	0	0	0	0	0	0
Fraction Csp3	1.00	0.50	0.00	0.33	1.00	0.17	1.00	0.33	1.00	0.94	0.94	0.74	0.95	0.95
Num. rotatable bonds	1	2	1	5	0	2	1	6	0	15	14	15	17	17
Num. H-bond acceptors	2	3	2	0	2	3	2	0	5	2	2	2	2	2
Num. H-bond donors	0	0	0	0	0	1	0	0	3	0	1	0	0	0
Molar refractivity	28.28	22.83	24.10	45.19	31.01	30.22	50.00	52.78	32.38	85.12	80.80	93.78	94.73	94.73
TPSA (Å²)	67.82	43.37	30.21	50.60	18.46	50.44	32.67	75.90	79.15	26.30	37.30	26.30	26.30	26.30
Lipinski’s rule	Yes	Yes	Yes	Yes	Yes	Yes	Yes	Yes	Yes	Yes	Yes	Yes	Yes	Yes
Bioavailability score	0.55	0.55	0.55	0.55	0.55	0.55	0.55	0.55	0.55	0.55	0.55	0.55	0.55	0.55
Pharmacokinetics/Toxicity prediction														
GI absorption	High	High	High	High	High	High	High	High	High	High	High	High	High	High
BBB permeant	Yes	No	Yes	Yes	Yes	No	Yes	Yes	No	Yes	Yes	No	No	No
P-gp substrate	No	No	No	No	No	No	No	No	Yes	No	No	No	No	No
CYP1A2 inhibitor	No	No	No	No	No	No	No	No	No	Yes	Yes	Yes	Yes	Yes
CYP2C19 inhibitor	No	No	No	No	No	No	No	No	No	No	No	No	No	No
CYP2C9 inhibitor	No	No	No	No	No	No	No	No	No	No	Yes	Yes	No	No
CYP2D6 inhibitor	No	No	No	No	No	No	No	No	No	No	No	No	No	No
CYP3A4 inhibitor	No	No	No	No	No	No	No	No	No	No	No	No	No	No
AMES toxicity	Yes	No	No	No	No	No	No	No	No	No	No	No	No	No
Hepatotoxicity	No	No	No	No	No	No	No	No	No	No	No	No	No	No
hERG I/II inhibitors	No	No	No	No	No	No	No	No	No	No	No	No	No	No

* Compound name is the same as listed in Table 1.

**Table 8:** Review of the phytochemical compounds identified in *A. subhirsutum* L. (bulbs and aerial parts) extracts from different origins.

Organ Tested/Origin	Solvent and Technique Used	Identified Molecules	Reference
Fresh bulbs (Hail, Saudi Arabia)	Methanol HR-LCMS **	Tripeptides: Tyr Trp Phe, Asn Asn Asn, Cys Tyr Trp, Thr Asp Asn, Cys Tyr Trp, Phe Glu, Asp Arg Tyr, Val Ser Cys, Asn Gln Ala, Val Glu Asp, Gly Tyr Lys, Lys Arg Lys; Dipeptides: Pro Leu, His Asp, Glu Thr, Phe Pro. Bioactive compounds: methyl *N*-(amethylbutyryl) glycine; bis(2-hydroxypropyl)amine; cepharanthine; 2-methylene-5-(2,5dioxotetrahydrofuran-3-yl)-6-oxo--10,10-dimethylbicyclo [7:2:0] undecane; (22S)-1alpha,22,25-trihydroxy-26,27-dimethyl-23,23,24,24-tetradehydro-24ahomovitamin D3/(22S)-1al; L-4-hydroxy-3-methoxy- amethylphenylalanine *N*-(2-fluro-ethyl) arachidonoyl amine; 1-nonadecanoyl-2- (5Z,8Z,11Z,14Z,17Zeicosapentaenoyl)-sn-glycerol; TG(16:1(9Z)/17:2(9Z,12Z)/20: 5(5Z,8Z,11Z,14Z,17Z)) [iso6]; L-4-hydroxy-3-methoxy-amethylphenylalanine; 11 alpha-acetoxykhivorin; methyl gamboginate; dihydrodeoxystreptomycin; 6 alpha-hydroxy castasterone; C16 sphinganine; 3beta,7alpha,12alpha-trihydroxy-5alpha-cholestan-26-oic acid; 4-oxomytiloxanthin; sebacic acid; tuberonic acid; 6-deoxocastasterone; linolenoyl lysolecithin; 19-amino-16-hydroxy-16-oxido-10-oxo-11,15,17-trioxa-165-phosphanonadecan-13-ylundecanoate GPETn(10:0/11:0) [U]; 3beta,7alpha,12alpha-trihydroxy-5alpha-cholestan- 26-oic acid, *N*-(2-hydroxyethyl) stearamide; 2,2-difluoro-hexadecanoic acid.	[16]
Dried pulverized flowering aerial parts/Bulbs (Palermo, Italy)	Absolute ethanol (≥99.8%) LC-ESI-MS^n+^	Sulfur compounds: allicin, gamma-glutamyl (S)-allylcysteine, gamma-glutamyl-S-methylcysteine, gamma-glutamyl-S-trans-propenyl cysteine, alliin, cycloalliin. Flavonoids and polyphenols: methoxy quercetin trisaccharide isomer, methoxy quercetin isomer, quercetin, methoxy quercetin trisaccharide isomer, luteolin, methoxy quercetin isomer, glucosyl gallate, kaempferol, methoxy quercetin isomer, 3,7-dimethylquercetin, tamarixetin (3,30,5,7-Tetrahydroxy-40-methoxyflavanone), 5,3´,4´-T-trihydroxy-3-methoxy-6,7-methylenedioxy flavone. Amide phenylpropanoid derivatives: coumaroyl-tyramine, *N*-trans-feruloyl-tyramine, coumaroyl-octopamine, *N*-trans-feruloyl-3-methoxytyramine.	[17]
Air-dried powdered aerial parts/bulbs Menderes (İzmir/Turkey)	Methanol LC-ESI-MS^n^	Phenolic compounds: benzoic acid; 3-hydroxybenzoic acid; 4-hydroxybenzoic acid; *p*-coumaric acid; vanillic acid; gallic acid; ferulic acid; syringic acid; aidzein; chrysin; kaempferol; luteolin; fisetin; morin; quercetin; 3-O-methylquercetin; isorhamnetin; galangin; myricetin; vitexin; hesperidin; 3-hydroxyflavone; naringenin; genistein; phenyl acetate; catechol; (+)-catechin; (−)-epicatechin; (−)-epigallocatechin gallate.	[18]

LC-ESI-MSn: liquid chromatography electrospray ionization tandem mass spectrometric; HR-LCMS **: high-resolution liquid chromatography mass spectroscopy.

**Table 9:** Review of the distribution of some identified bioactive compounds in some *Allium* plant species.

Bioactive Molecule	*Allium* Species/Variety	References
Methyl methanethiolsulfonate	*Allium hirtifolium*	[25]
	*Allium hooshidaryae*	[26]
	*Allium sativum*	[27]
	*Allium ursinum*	[28]
Furfural	*Allium fistulosum*	[29]
	Black garlic	[30]
Diallyl disulfide	*Allium hookeri*	[31]
	Black garlic	[30,32]
	*Allium sativum*	[27]
	*Allium tuncelianum*	[33]
2,4,5-trimethyl-1,3-dioxolane	*Allium hirtifolium*	[25]
5-hydroxymethylfurfural	*Allium hirtifolium*	[25]
	Black garlic	[32,34]
	*Allium fistulosum*	[29]
	*Allium sativum* (varieties Taicangbaip, Hongqixing, Ershuizao and single clove)	[35]
Trisulfide, di-2-propenyl	*Allium hookeri*	[31]
	Black garlic	[30]
	*Allium sativum* (varieties Taicangbaip, Hongqixing, Ershuizao and single clove)	[35]
	*Allium tuncelianum*	[33]
Beta-D-fructofuranosyl alpha-D-glucopyranoside	*Allium sativum* (varieties Taicangbaip, Hongqixing, Ershuizao and single clove)	[35]
Palmitic acid, methyl ester	*Allium sativum* (varieties Taicangbaip, Hongqixing, Ershuizao and single clove)	[35]
n-hexadecanoic acid	*Allium hirtifolium*	[25]
	*Allium fistulosum*	[29]
	*Allium willeanum*	[36]
	*Allium sativum* (varieties Taicangbaip, Hongqixing, Ershuizao and single clove)	[35]
9,12-octadecadienoic acid, methyl ester	*Allium hirtifolium*	[25]
	*Allium ampeloprasum*, var. *holmens*	[37]
Octadecanoic acid, methyl ester	*Allium fistulosum*	[29]

## Data Availability

The data generated and analyzed during this study are included in this article.

## Supplementary Materials

The following supporting information can be downloaded at: https://www.mdpi.com/article/10.3390/plants11040495/s1, Supplementary Material S1: Phytoconstituents identified in *A. subhirsutum* L. aqueous extract using GC-MS technique.
