# Supplementary material for: Phytochemical Profiling of Allium subhirsutum L. Aqueous Extract with Antioxidant, Antimicrobial, Antibiofilm, and Anti-Quorum Sensing Properties: In Vitro and In Silico Studies
Source: Plants (Basel). 2022 Feb 11;11(4):495. doi: 10.3390/plants11040495 (PMC8878528; doi:10.3390/plants11040495)
Supplement: Supplementary file 1 [file plants-11-00495-s001.zip › plants-1584717-supplementary.pdf]

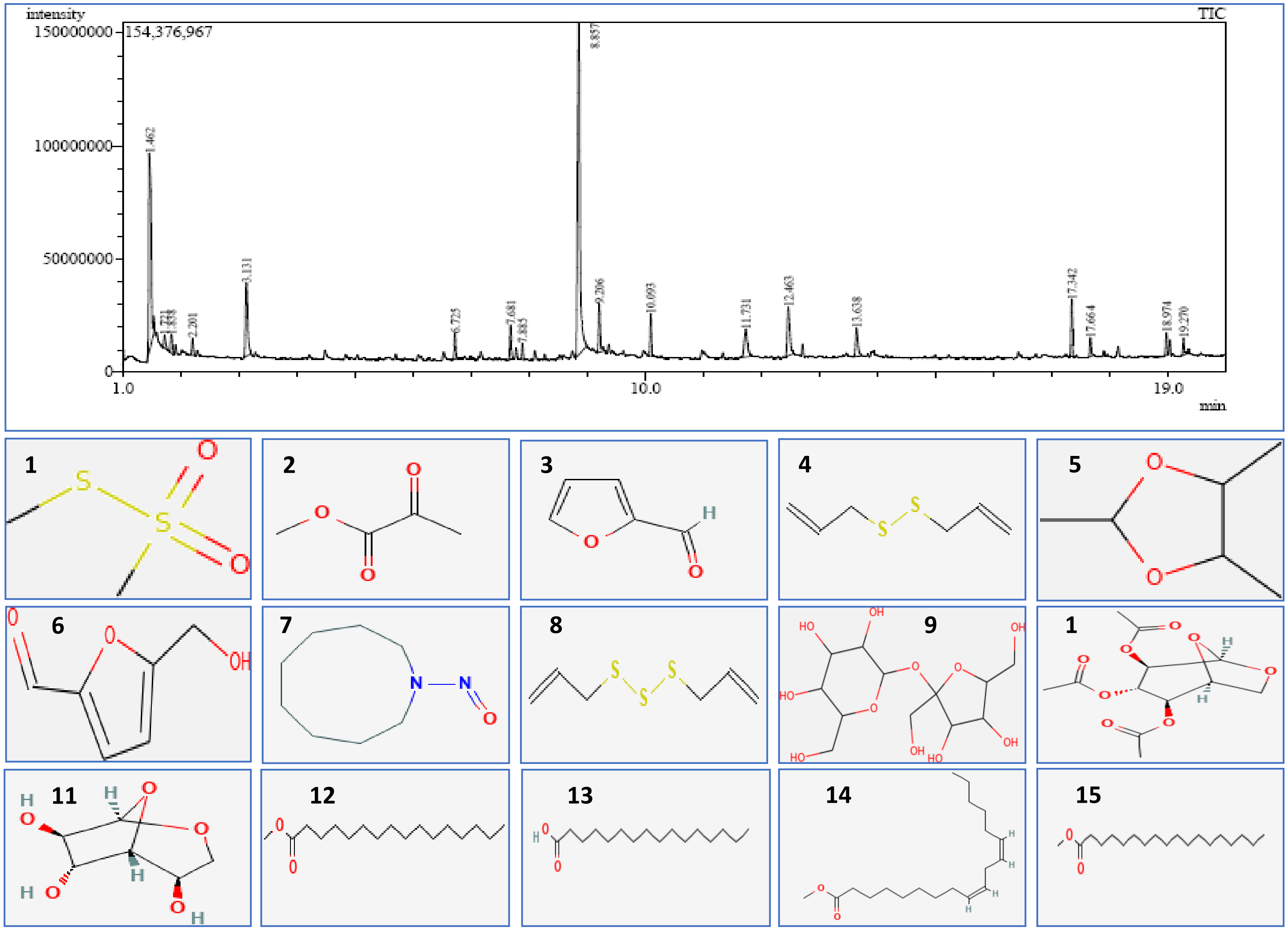

Supplementary material S1. Phytoconstituents identified in *A. subhirsutum* L. aqueous extract using GC-MS technique. Compounds identified: 1: Methyl methanethiolsulfonate; 2: Propanoic acid, 2-oxo-, methyl ester; 3: Furfural; 4: Diallyl disulphide; 5: 2,4,5-Trimethyl-1,3-dioxolane; 6: 5-Hydroxymethylfurfural; 7: 1H-Azonine, octahydro-1-nitroso-; 8: Trisulfide, di-2-propenyl; 9: Beta-D-fructofuranosyl alpha-D-Glucopyranoside; 10: Beta-D-Glucopyranose, 1,6-anhydro-; 11: Beta-D-glucofuranose, 1,6-Anhydro-; 12: Palmitic acid, methyl ester; 13: n-Hexadecanoic acid; 14: 9,12-Octadecadienoic acid, methyl ester; 15: Octadecanoic acid, methyl ester.
